# Letter to the editor regarding a global overview of sickle cell disease: populations, policy limitations and urgent need for comprehensive SCD clinical care, a systematic review

**DOI:** 10.1093/jscdis/yoaf034

**Published:** 2025-10-10

**Authors:** Enrico M Novelli

**Affiliations:** Division of Classical Hematology, Department of Medicine, University of Pittsburgh, Pittsburgh, PA 15213, United States

**Keywords:** Sickle cell disease, health inequities, newborn screening, global health, stigma and discrimination, policy and innovation

In their article titled “A Global Overview of Sickle Cell Disease: Populations, Policy limitations and urgent need for comprehensive SCD clinical care, A Systematic Review,”^1^ Joseph et al comprehensively report the status of sickle cell disease (SCD) worldwide and offer a plan to advance the field and correct SCD’s long history of neglect.

This is important work, as the SCD landscape outside the United States has traditionally been underrepresented in scientific literature and public discourse. The history of SCD is intrinsically linked to broader struggles for equity and human rights. In the United States, the seminal book by Keith Wailoo, *To Die in the City of Blues* (2001),^2^ remains a poignant reminder of how SCD epitomized the fight for justice by exposing racial inequities in health care, the politics of federal funding, and the stigmatization of Black communities. Yet, as one broadens the horizon to SCD worldwide, the universality of these themes emerges clearly. Across continents, individuals living with SCD often find themselves at the bottom of social hierarchies. In Africa, where the disease burden is greatest, most children with SCD are either invisible to the health care system as they die before diagnosis or live under stigma—sometimes ascribed to myths such as *ogbanje*, which portray affected children and families as cursed. In Europe, SCD disproportionately affects migrants from Africa who already face marginalization and limited access to social support. In South Asia, SCD is most common among Scheduled Tribes and other impoverished groups living in the so-called “sickle belt.”

The most obvious result of SCD’s neglect is its unacceptably high mortality in the 21^st^ century. In sub-Saharan Africa, SCD remains one of the leading causes of under-5 mortality and in the United States where universal newborn screening (NBS) has been in place for decades—adult mortality remains poor. The unfavorable comparison Joseph et al^1^ make between SCD and cystic fibrosis (CF) is corroborated by mortality trends in the 2 diseases: a 10-fold funding differential has resulted in dramatic survival gains in CF, with mean age of death rising from 30 to 48 years between 2008 and 2023, as compared to a rise from 40 to 46 years in SCD, which translates into an age-adjusted mortality rate of 0.15 per 100 000 for CF versus 0.29 for SCD.^3^

For hematologists, the American Society of Hematology (ASH) Annual Meeting has been a painful reminder of the disparity in innovation between SCD and other genetic diseases. Until only a decade ago, abstracts on SCD therapy predictably revolved around hydroxyurea or transfusion strategies, while hemophilia research, buoyed by public and industry investment, was advancing rapidly with novel biologicals, extended half-life factors, and gene therapy. The contrast was disheartening.

There is, however, a sense that the tide has finally turned. From an orphan disease, SCD has become a darling of Pharma, with many drugs in development and 2 gene therapies approved by the Food and Drug Administration (FDA). Yet translating decades of preclinical work in sickle mice into new therapeutics has proven challenging. This was highlighted by disappointing results in global trials of crizanlizumab^4^ and the market withdrawal of voxelotor,^5^ also based on worrisome safety signals in global trials. The lesson from these experiences is clear: future approaches to new therapies must be global at inception and adopt biomarkers and endpoints that are relevant across diverse populations. North–South collaborations and frugal or reverse innovation—where new treatments are piloted in sub-Saharan Africa simultaneously with high-income countries—should be the new model. This approach also ensures that affordability, local production strategies, discounted drug schemes and effective drug distribution and delivery channels are integrated in the drug development process from the outset. In other words, the fulcrum of research needs to shift south.

Still, progress in SCD cannot occur without first identifying children born with the disease, and so NBS remains the cornerstone of any far-reaching SCD policy. The ASH Consortium on Newborn Screening in Africa (CONSA)^6^ and India’s gargantuan National Sickle Cell Anaemia Elimination Mission (NSCAEM)^7^ are the most ambitious efforts in screening and diagnosis to date in low- and middle-income countries. CONSA is a demonstration project designed to spark progress in NBS by engaging key stakeholders and generating momentum that can eventually translate into government action. It leverages ASH’s convening power to bring together international leaders in SCD from 7 sub-Saharan African Countries, develop concerted protocols for NBS and early intervention strategies, and generate data that can drive policy change. NSCAEM is a large-scale government initiative launched in 2023 to screen, diagnose, and provide free care and counseling for individuals with SCD—particularly in tribal and high-prevalence regions—with the goal of eliminating the disease burden by 2047. If successful, it could serve as a model for other nations and demonstrate the impact that decisive government action can have in alleviating the burden of SCD. Yet the challenges of large-scale NBS—including training of health care providers and community health workers, addressing cultural bias related to SCD and NBS, timely return of results, engagement and long-term retention in care—are immense. If CONSA can successfully overcome the pitfalls of relying on external funding without sustained government adoption and NSCAEM can avoid the risks of coercion by social pressure (eg, invasive marriage counseling), and stigmatization through genetic status cards handed to participants, both programs can effect transformative change worldwide.

Major transnational efforts must continue, but each country also needs its own comprehensive SCD agenda. The NASEM report in the United States.^8^ and the *Lancet Haematology* Commission^9^ provide roadmaps that should guide national policy. Together with Joseph et al.’s systematic review,^1^ these white papers emphasize that training of health workers and providers must be central to any comprehensive program.

The challenges ahead may seem insurmountable, given the pervasive stigma and discrimination faced by people with SCD. Yet the transformation of HIV/AIDS—from a near-untreatable plague to a chronic disease within two decades—shows what is possible with political will and funding. There is no reason SCD cannot undergo the same transformation—if, and only if, the necessary investments are made.
